# *MTHFR* polymorphism's influence on the clinical features and therapeutic effects in patients with migraine: An observational study

**DOI:** 10.3389/fneur.2022.1074857

**Published:** 2022-12-23

**Authors:** Jianhao Guo, Xing Hao, Rongrong Wang, Ke Lian, Jun Jiang, Na Chen, Zhiying Feng, Yuefeng Rao

**Affiliations:** ^1^The First Affiliated Hospital, Zhejiang University School of Medicine, Hangzhou, China; ^2^Hangzhou Dunen Medical Laboratory Co., Ltd., Hangzhou, China

**Keywords:** migraine, folic acid, individualized medicine, MTHFR polymorphism, therapeutic efficacy

## Abstract

**Objective:**

Our study aimed to evaluate the influence of methylenetetrahydrofolate reductase (*MTHFR*) polymorphism on the clinical features and therapeutic effects in patients with migraine.

**Methods:**

The data of 135 patients with migraine were collected from January 2021 to December 2021. The *MTHFR* C677T polymorphism was analyzed. The pain intensity was evaluated using a numerical rating scale (NRS) during treatment. The levels of folic acid, homocysteine (Hcy), vitamin B12, interleukin-2 (IL-2), IL-4, and ferritin, and changes of NRS were compared between folic acid and conventional treatment groups stratified by different genotypes of *MTHFR* in migraine patients.

**Results:**

The levels of Hcy and ferritin in male patients were higher than that in female patients (*P* < 0.05); Compared with CC and CT genotype groups, the TT genotype group showed significantly higher Hcy levels (*P* < 0.05) and lower folic acid levels (*P* < 0.05); In both folic acid and conventional treatment groups, a significant decrease in NRS score was observed in different genotypes post-treatment (*P* < 0.05). Patients with TT genotype in the folic acid treatment group showed better therapeutic efficacy than conventional treatment group (*P* < 0.05). There is no significant difference in the therapeutic efficacy in other genotypes between the two groups (*P* > 0.05).

**Conclusion:**

The *MTHFR* C677T genotyping may provide a new method to guide and optimize individualized medication for migraine patients.

## Introduction

Migraine is a chronic, multifactorial neurovascular disease that is typically characterized by recurring and disabling headache attacks, as well as an impaired autonomic nervous system (1). Symptoms associated with migraine include paroxysmal unilateral throbbing pains, nausea, vomiting, photophobia and phonophobia (2). The incidence of migraine is about 15% worldwide, the prevalence rate is 9.3% in China, and the ratio of males to females is about 1:3 (3, 4).

The pathogenesis of migraine is complex and remains unclear. Migraine is a multifactorial disorder with pathogenesis influenced by dietary, psychological, environmental, and genetic factors. Except for symptomatic treatments, no special drugs are available for migraine. The first-line therapeutic drugs for migraine include triptans, non-steroidal anti-inflammatory drugs, antiemetics, and so on. A combination strategy is commonly used to reduce the degree of the headache and control other symptoms; however, it is related to medication-overuse headaches, adverse reactions, and substantial financial burdens (5, 6), so the treatment discontinuation rate is high. Previous studies reported that 66.7% of patients discontinued their medications (7). Therefore, a significant need exists for other effective, safe, and affordable treatments at present.

Methylenetetrahydrofolate reductase (MTHFR) is a crucial enzyme for homocysteine (Hcy) metabolism and regulates Hcy level *in vivo*, mainly by affecting folic acid absorption and metabolism (8, 9). MTHFR catalyzes the 5,10-methylenetetrahydrofolate into 5-methyltetrahydrofolate (CH_3_-THF) to provide the carbon in the metabolism process of Hcy. The *MTHFR* 677C → T polymorphism reduces MTHFR enzyme activity, resulting in hyperhomocysteinemia (HHcy). HHcy has become a well-established healthy risk factor associated with neurological disorders, cognitive decline, and cardiovascular disease. HHcy may increase neuronal excitability and release many headache mediators and other inflammatory factors; finally, it induces migraine (10–12). Supplementation of folic acid can reduce the level of Hcy, which has been used to prevent and treat other diseases related to Hcy (13, 14). Several clinical studies preliminarily suggested that targeted folic acid supplementation for migraine patients can help prevent the onset and treatment of migraine (10, 15). Some people may respond better to folic acid supplementation, but the conclusions are inconsistent (16). The *MTHFR* 677C → T variant can decrease the activity of MTHFR, reduce the tetrahydrofolate levels intracellularly, increase the folic acid demand, and counteract the folic acid supplement (9). However, limited studies about the influence of *MTHFR* C677T polymorphism on migraine's clinical features and effects from different treatments have been carried out, such as folic acid. Thus, this article is aimed to determine the role of *MTHFR* polymorphism in the treatment of migraine and the optimization of individualized medication for migraine patients.

## Materials and methods

### Study design

This is a retrospective study using the detection of the *MTHFR* polymorphism to evaluate the efficacy of patients who received treatments for migraine between January 2021 and December 2021 in the First Affiliated Hospital of Zhejiang University (FAHZJ), a hospital located in the Zhejiang province, China. A total of 135 patients diagnosed with migraine by physicians were enrolled in our hospital. Patients received folic acid (5 mg), or conventional drugs for migraine treatment were eligible. Ethical approval was obtained from the authorizing ethics committee of FAHZJ (IIT20220564A). The data were anonymous, and the requirement for informed consent was waived for our retrospective study.

### *MTHFR* genotyping and laboratory testing

Genomic DNA was prepared from peripheral blood samples by cell lysis assay. For each patient, 2 mL of peripheral venous blood was collected with EDTA as an anticoagulant for testing *MTHFR* C677T genotypes. A test kit (PCR and microarray method, Shanghai Bio Science and Technology Co., Ltd., China) was used to identify the *MTHFR* C677T genotypes as follows: CC genotype (heterozygous, fast metabolizer), CT genotype (mutant, intermediate metabolizer) and TT genotype (homozygous mutant, slow metabolizer). The serum analytes measured were folic acid, Hcy, ferritin, vitamin B12, interleukin-2 (IL-2), IL-4, IL-6, IL-10, TNF-α, IFN-γ, and IL-17A.

### Efficacy assessment

The treatment duration was 1 month. The pain intensity was evaluated using a numerical rating scale (NRS) ranging from 0 to 10. Higher scores indicated greater pain: mild pain (NRS 1–3), moderate pain (NRS 4–6), and severe pain (NRS 7–10). Furthermore, changes in NRS scores were determined by comparing the NRS scores before and after treatment.

### Statistical analysis

Data were statistically processed and analyzed by SPSS version 26.0. Measurement data were presented as mean ± standard deviation (SD) and were compared with *t*-test or One-Way ANOVA. Enumeration data were presented as percentages. A two-tailed *P* < 0.05 demonstrated statistically significant differences.

## Results

### Clinical characteristics

Clinical characteristics are shown in Table 1 and Supplemental Table 1. Among the 135 migraine patients, 43 were males, and 92 were females, ranged from 13 to 69 years old (mean and median age, 35.2 and 34 years old, respectively). Based on gene detection, 35 patients (25.92%) were fast metabolizer (CC genotype), 72 patients (53.33%) were intermediate metabolizer (CT genotype), and 28 patients were (20.74%) slow metabolizer (TT genotype). The conventional drugs used were listed in Table 1 and mainly included non-steroidal anti-inflammatory drugs (acetaminophen, imrecoxib, celecoxib, diclofenac diethylamine), muscle relaxant (tizanidine, eperisone), methylcobalamin, pregabalin, gabapentin, and topiramate.

**Table 1:** Clinical characteristics of migraine patients at baseline.

**Variables**		**N (%) or (Mean ±SD)**
Age (years)		35.20 ± 12.64
Gender	Male	43 (31.9)
	Female	92 (68.1)
NRS	Mild (0~3)	52 (38.5)
	Moderate(4~6)	70 (51.9)
	Severe (7~10)	13 (9.62)
Pain sensation	Persistent	21 (15.6)
	Paroxysmal	6 (4.4)
	Pulsatile	18 (13.3)
	Splitting	4 (3.0)
	Stabbing	3 (2.2)
Pain location	Bilateral	54 (40.0)
	Unilateral	81 (60.0)
	Temporal	16 (11.9)
	Occiput	59 (43.7)
	Parietal	55 (40.7)
	Frontal	47 (34.8)
	Neck-Shoulder	30 (22.2)
	Temple	61 (45.2)
	Periocular	35 (25.9)
Tenderness	Occiput	23 (17.0)
	Temporal	23 (17.0)
	Occipital nerve	21 (15.6)
	Parapophyses of cervical	27 (20)
	Trapezius	29 (21.5)
	Sternocleidomastoid	1 (0.7)
	Neck-Shoulder	15 (11.1)
Pain duration (years)	0~5	50 (37.0)
	6~10	40 (29.6)
	11~15	12 (8.9)
	16~20	20 (14.8)
	20~	13 (9.6)
Duration of increased pain (months)	0~1	11 (8.1)
	2~5	13 (9.6)
	6~12	16 (11.9)
	12~	10 (7.4)
Pain frequency	Once a month	23 (17.0)
	Twice a month	57 (42.2)
	Thrice a month	44 (32.6)
	More than thrice a month	11 (8.1)
Family history for migraine		9 (6.7)
Analgesic drugs	Tizanidine	105 (77.8)
	Methylcobalamin	60 (44.4)
	Folic acid	45 (33.3)
	Qing Peng Ruan Gao	38 (28.1)
	Du Liang Ruan Jiao Nang	37 (27.4)
	Pregabalin	35 (25.9)
	Acetaminophen	34 (25.2)
	Eperisone	34 (25.2)
	Calcitriol	30 (22.2)
	Imrecoxib	26 (19.3)
	Gu Tong Tie Gao	25 (18.5)
	Gabapentin	24 (17.8)
	Topiramate	19 (14.1)
	Promethazine	16 (11.9)
	Flunarizine	15 (11.1)
	Lidocaine	14 (10.4)
	Loxoprofen	13 (9.6)
	Ropivacaine	12 (8.9)
	Botulinum toxin	12 (8.9)
	Vitamin B6	12 (8.9)
	Bai Mai Ruan Gao	9 (6.7)
	Tandospirone	8 (5.9)
	Propacetamol	8 (5.9)
	Celecoxib	7 (5.2)
	Diclofenac diethylamine	7 (5.2)
	Adenosylcobalamin	7 (5.2)

### Serum indexes in different genders

Serum indexes are shown in Table 2. Serum levels of Hcy and ferritin were significantly higher in males than those in females (*P* < 0.05). There was no significant difference between genders on the levels of vitamin B12, IL-2, IL-4, IL-6, IL-10, TNF-α, IFN-γ, and IL-17A (*P* > 0.05).

**Table 2:** Comparison of serum indexes in different genders (mean ± SD).

	**Male** **(*n* = 43)**	**Female** **(*n* = 92)**	***t***	***P*-value**
Folic acid	7.106 (6.09)	9.704 (12.80)	1.350	0.18
Vitamin B12	468.9 (273.0)	739.2 (972.2)	1.809	0.072
Ferritin	224.4 (192.5)	68.23 (61.5)	7.375	<0.0001
Hcy	13.94 (9.671)	7.764 (3.074)	5.691	<0.0001
IL-2	0.686 (1.420)	1.091 (1.676)	0.412	0.681
IL-4	1.232 (1.481)	1.853 (5.790)	0.684	0.4952
IL-6	2.464 (1.803)	2.818 (2.812)	0.747	0.456
IL-10	1.133 (0.962)	1.245 (1.573)	0.431	0.667
TNF-α	3.243 (5.040)	3.863 (6.750)	0.530	0.596
IFN-γ	3.733 (4.526)	6.240 (19.400)	0.826	0.41
IL-17A	7.954 (20.470)	7.657 (19.970)	0.060	0.947

### Serum indexes in different genotypes of *MTHFR* C677T

**Figure 1:**
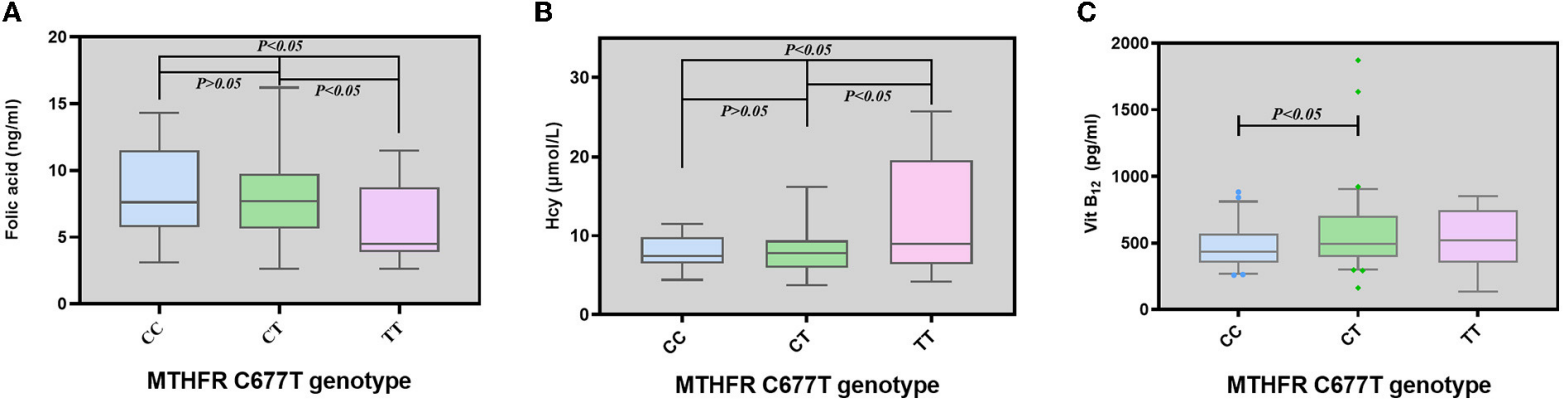
Levels of folic acid, homocysteine, and vitamin B12 among different genotypes of *MTHFR* in migraine patients. **(A)** Levels of folic acid among migraine patients with different genotypes of *MTHFR*. **(B)** Levels of Hcy among migraine patients with different genotypes of *MTHFR*. **(C)** Levels of vitamin B12 among migraine patients with different genotypes of *MTHFR*. Horizontal lines represent median values, box represent the quartiles, and whiskers represent the range of data.

Summary data are shown in Figure 1 and Table 3. Data analysis showed lower folic acid levels and higher Hcy levels in the TT genotype group (*P* < 0.05). There was a significant difference in vitamin B12 levels between the CC and CT genotype groups (*P* < 0.05, Figure 1). No significant difference was found on the levels of ferritin, IL-2, IL-4, IL-6, IL-10, TNF -α, IFN-γ, and IL-17A among the three genotype groups (*P* > 0.05).

**Table 3:** Comparison of serological indicators in migraine patients with different *MTHFR* genotypes (Mean ± SD).

**Serological indicators**	**C/C** **(*n* = 35)**	**C/T** **(*n* = 72)**	**T/T (*n* = 28)**
Ferritin	100.9 ± 99.37	117.2 ± 128.8	85.79 ± 73.83
IL-2	0.90 ± 0.86	0.92 ± 0.91	0.83 ± 0.79
IL-4	1.33 ± 1.49	1.22 ± 1.2	1.23 ± 1.55
IL-6	2.91 ± 2.64	2.70 ± 2.76	2.40 ± 1.70
IL-10	1.48 ± 2.10	1.08 ± 0.91	1.10 ± 0.87
TNF-α	3.65 ± 5.11	3.90 ± 7.11	4.68 ± 7.46
TNF-γ	4.39 ± 6.36	2.91 ± 3.29	3.86 ± 4.57
IL-17A	9.35 ± 25.27	7.20 ± 18.58	6.62 ± 14.28

CC genotype: homozygous; CT genotype: heterozygous; TT genotype: homozygous mutation.

### Treatment response by *MTHFR* C677T genotype

**Figure 2:**
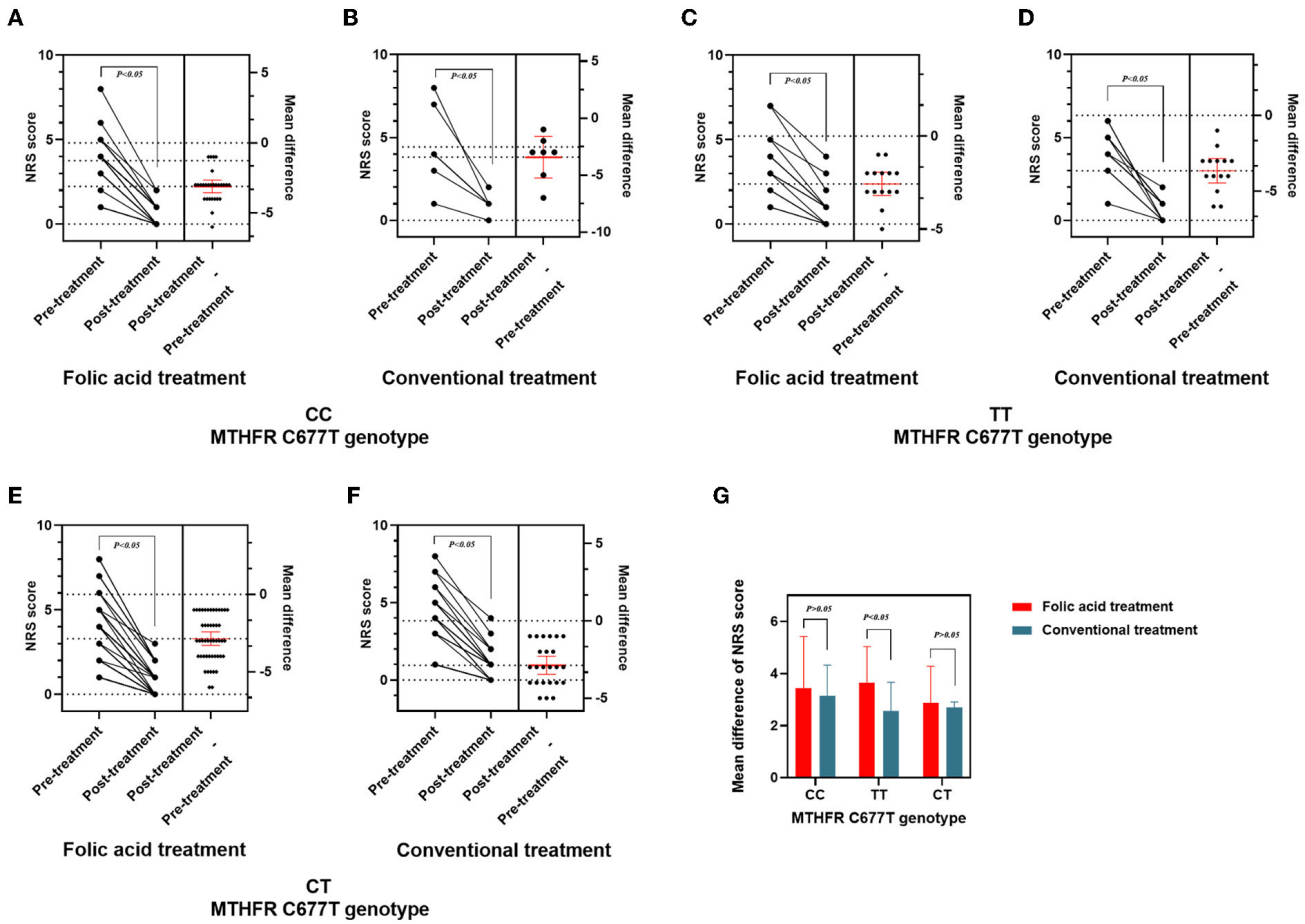
Therapeutic effects for migraine patients with different *MTHFR* genotypes before and after treatment. **(A–F)** NRS score and changes of NRS in patients with different genotypes of *MTHFR* before and after treatment. **(G)** Mean difference of NRS score between folic acid and conventional treatment groups stratified by different genotypes of *MTHFR* in migraine patients.

The NRS score variations during treatment are shown in Figure 2 and Table 4. A significant decrease in NRS score was observed post-treatment (*P* < 0.05). Treatment with folic acid was effective in three genotype groups (*P* < 0.05). Patients with TT genotype in the folic acid treatment group showed better therapeutic efficacy than conventional treatment group (*P* < 0.05). There is no significant difference in the therapeutic efficacy in other genotypes between the two groups (*P* > 0.05).

**Table 4:** Comparison of treatment response by *MTHFR* C677T genotype.

***MTHFR* C677T genotype**	**NRS**	**Folic acid treatment group**	**Conventional treatment group**	***t***	***P* ^1^**
	NRS pre-treatment	4.29 ± 1.33	3.64 ± 1.91	1.036	0.309
TT	NRS post-treatment	0.64 ± 0.63	1.07 ± 1.21	1.177	0.25
	Variation of NRS pre-and post-treatment	3.64 ± 1.39	2.57 ± 1.09	2.267	0.0319
	*n*	14	14		
	NRS pre-treatment	3.83 ± 2.12	3.33 ± 2.19	0.932	0.354
CT	NRS post-treatment	0.96 ± 1.04	0.65 ± 0.80	1.428	0.156
	Variation of NRS pre-and post-treatment	2.88 ± 1.40	2.69 ± 0.22	0.623	0.6225
	*n*	24	48		
	NRS pre-treatment	4.429 ± 2.37	3.75 ± 1.58	0.918	0.365
CC	NRS post-treatment	1.00 ± 0.57	0.64 ± 0.62	1.377	0.177
	Variation of NRS pre-and post-treatment	3.43 ± 1.99	3.14 ± 1.18	0.497	0.623
	*n*	7	28		

1 Comparisons were performed between folic acid treatment group and conventional treatment group among different *MTHFR* C677T genotypes at different time point.

## Discussion

Migraine is a neurovascular disorder characterized by recurrent moderate to severe headache, usually near the hemicranial head and often accompanied by vomiting, diarrhea, photophobia, and fear of sound. If one parent has migraine, the children are about 40 percent more likely to have migraine (17). Moreover, while both parents have migraines, the risk of migraine (17) for their children is up to 75%.

The MTHFR enzyme encoded by the *MTHFR* gene is critical in metabolizing methionine folate. This enzyme reduces 5,10-methylene tetrahydrofolate to 5-methyl tetrahydrofolate (5-MTHF) (8). As a methyl donor, 5-MTHF can maintain the balance of Hcy through the methylation of Hcy in the blood (18). Inadequate consumption of folic acid, or abnormality of the enzymes involved in folic acid metabolism, can lead to decreased levels of folic acid. Thus, the methionine production can be inhibited, resulting in an accumulation of Hcy in the body and HHcy. At the same time, the increased Hcy level contributes to the development of migraine through a series of complex events. A high level of Hcy may impair methylation reactions, which disrupts the normal functions of blood vessels and nerves, inhibit the activity of the cerebral cortex, and decrease local blood flow (19). These reactions can further increase the inflammatory cytokines (such as 5-HT, IL-6, and TNF-α) in cerebral microvascular endothelial cells (20), and finally lead to migraine.

Recently, numerous studies have demonstrated that *MTHFR* polymorphism is associated with migraine and serum Hcy concentrations (21). The genetic polymorphism of the *MTHFR* can cause a decrease in MTHFR enzyme activity. Compared with the CC genotype, the CT and the TT genotypes show 65% and 30% of MTHFR enzyme activities, respectively (22). Decreased folic acid levels were observed in patients with the slow metabolism TT genotype. Wang et al. studied the genetic susceptibility of C677T polymorphism to migraine in Asians (23). They suggested that the increase of Hcy may be involved in the pathogenesis of migraine and folic acid, Hcy serum level, and *MTHFR* polymorphism are related to the occurrence of migraine (23). Some studies showed that the Hcy level in patients with migraine is significantly higher than that of the control group, especially during migraine attacks (24). Our study compared the serum folate and Hcy levels among migraine patients with different genotypes. The result showed that the serum folate levels of patients with slow and intermediate metabolic types were significantly lower than those with fast metabolic types. This result is consistent with the data reported in previous literature about the mechanism of MTHFR enzymes.

The study of Pan et al. showed that (25) the vitamin B12, folic acid, and Hcy levels in the migraine group were negatively correlated, indicating that the elevation of Hcy level may be related to the inducement of migraine and that supplementation of folic acid and vitamin B12 may prevent migraine. Lea et al. (26) suggested that vitamin supplements (2 mg folic acid, 25 mg vitamin B6, and 400 micrograms of vitamin B12 every day) and lower Hcy levels can effectively reduce headache frequency and pain severity in 52 migraine patients with aura. Menon et al. (15) treated 206 migraine patients with vitamin supplements. The results showed that vitamin supplements effectively reduced Hcy levels and headache severity in migraine patients. The C allele of the *MTHFR* C677T variant has more reductions in Hcy levels and migraine pain severity compared with the TT genotype. Stratified analysis by the genotype of the vitamin treatment group, *MTHFR* genotype variation affects the therapeutic effect of migraine patients. In our study, migraine patients with CT and TT genotype were divided into a folic acid treatment group and a conventional treatment group. Our results showed that NRS decreased after treatment, with significant differences in numerical values of NRS before and after treatment. The pain and symptoms of migraine patients with the TT genotype were significantly relieved after treatment with folic acid supplementation. Besides, the curative effect of the TT genotype group treated with folic acid supplementation was better than that of conventional treatment group (*P* < 0.05). Fourteen migraine patients with high Hcy had a noticeable curative effect after taking medicine for 1 month. The doctors used folic acid and methylcobalamin to treat migraine the first time and then adjusted the treatment regimen until the above indexes returned to normal range according to the levels of folic acid, vitamin B12, and Hcy in serum. Meanwhile, the patients should pay attention to eating more foods rich in folic acid in their diet. After treatment, the patients' headache was significantly relieved, suggesting that the *MTHFR* C677T genotyping has a particular clinical value in guiding the individualized treatment of migraine. Apart from this, it affirms the curative effect of folic acid supplementation on migraine patients with high Hcy.

Some limitations still existe. In our study, 35 patients (25.92%) were fast metabolizer (CC genotype), 72 patients (53.95%) were intermediate metabolizer (CT genotype), and 28 patients were (20.74%) slow metabolizer (TT genotype). The result seems different from the data reported in public databases (27). However, in Chinese populations, geographical and ethnic variations were observed in *MTHFR* C677T genotypes. In a large sample that pooled the results of epidemiological studies on the distributions of *MTHFR* C677T genotypes in healthy populations living in Mainland China, the mean frequency of 677TT genotype was 20% (95% confidence interval: 18–23%) (28). The frequency of the 677T allele ranged from 24.0% in Hainan province and 63.1% in Shangdong province. Thus, the polymorphic distribution of *MTHFR* C677T in our study is within the reported range in previous studies among the Chinese population (28). In addition, this sample size of our study is relatively small, so potential confounders to migraine (such as age distribution, sex percentage, and pain duration) were not further checked *via* multivariate logistic regression. Therefore, the results of this study also need to be further verified with a well-designed and large sample study. In summary, this study has provided valuable information from real-world clinical practices about individualized medication for migraine treatments.

## Conclusion

In conclusion, *MTHFR* polymorphism may be beneficial to guide and optimize individualized medication for migraine treatments. In the future, the relationship between *MTHFR* polymorphism and migraine treatment can be further studied in larger sample populations with a well-controlled experimental design, especially for vitamin B supplementation.

## Data availability statement

The original contributions presented in the study are included in the article/Supplementary material, further inquiries can be directed to the corresponding author.

## Ethics statement

The studies involving human participants were reviewed and approved by the First Affiliated Hospital of Zhejiang University. Written informed consent for participation was not required for this study in accordance with the national legislation and the institutional requirements.

## Author contributions

Conception and design: JG, YR, and XH. Provision of study materials or patients: XH, NC, and RW. Collection and assembly of data: ZF, KL, and JJ. Data analysis and interpretation: KL. All authors contributed to the writing and final approval of manuscript.

## References

[1] Xuejun S Bifa F You W Daying Z Yan L Jisheng H. revision of IASP definition of pain. Chin J Pain Med. (2020) 26:641–4. 10.3969/j.issn.1006-9852.2020.09.001

[2] De Felice M Ossipov MH Wang R Dussor G Lai J Meng ID. Triptan-induced enhancement of neuronal nitric oxide synthase in trigeminal ganglion dural afferents underlies increased responsiveness to potential migraine triggers. Brain. (2010) 133:2475–88. 10.1093/brain/awq159 20627971 PMC3139937

[3] Shunwei L Yansheng L ruozhuo L Xiangyang Q Qi W Xiaosu Y. The Chinese guidelines for the diagnosis and treatment of migraine. Chin J Pain Med. (2011) 17:65–86. 10.3969/j.issn.1006-9852.2011.02.001

[4] Vos T Lim SS Bisignano C Cruz J Mirzaei-Alavijeh M. Global burden of 369 diseases and injuries in 204 countries and territories, 1990–2019—a systematic analysis for the Global Burden of Disease Study 2019. Lancet. (2020) 396:1204–22. 10.1016/S0140-6736(20)30925-9 33069326 PMC7567026

[5] Archibald N Lipscomb J McCrory DC AHRQ. Technical Reviews. Resource Utilization and Costs of Care for Treatment of Chronic Headache. Rockville, MD: Agency for Health Care Policy and Research (US). (1999). 20734516

[6] Wells RE O'Connell N Pierce CR Estave P Penzien DB Loder E. Effectiveness of mindfulness meditation vs headache education for adults with migraine: a randomized clinical trial. JAMA Intern Med. (2021) 181:317–28. 10.1001/jamainternmed.2020.7090 33315046 PMC7737157

[7] Hepp Z Dodick DW Varon SF Chia J Matthew N Gillard P. Persistence and switching patterns of oral migraine prophylactic medications among patients with chronic migraine: a retrospective claims analysis. Cephalalgia. (2017) 37:470–85. 10.1177/0333102416678382 27837173 PMC5405847

[8] van der Pol KH Nijenhuis M Soree B de Boer-Veger NJ Buunk AM Guchelaar HJ. Dutch pharmacogenetics working group guideline for the gene-drug interaction of ABCG2, HLA-B and Allopurinol, and MTHFR, folic acid and methotrexate. Eur J Hum Genet. (2022) 1–8. 10.1038/s41431-022-01180-0. [Epub ahead of print]. 36056234 PMC10853275

[9] Shaik MM Gan SH. Vitamin supplementation as possible prophylactic treatment against migraine with aura and menstrual migraine. Biomed Res Int. (2015) 469529. 10.1155/2015/469529 25815319 PMC4359851

[10] Askari G Nasiri M Mozaffari-Khosravi H Rezaie M Bagheri-Bidakhavidi M Sadeghi O. The effects of folic acid and pyridoxine supplementation on characteristics of migraine attacks in migraine patients with aura: a double-blind, randomized placebo-controlled, clinical trial. Nutrition. (2017) 38:74–9. 10.1016/j.nut.2017.01.007 28526386

[11] Liu L Yu Y He J Guo L Li H Teng J. Effects of MTHFR C677T and A1298C polymorphisms on migraine susceptibility: a meta-analysis of 26 studies. Headache. (2019) 59:891–905. 10.1111/head.13540 31045246

[12] Tietjen GE Collins SA. Hypercoagulability and migraine. Headache. (2018) 58:173–83. 10.1111/head.13044 28181217

[13] ChenHLiuSGeBZhouDLiMLiW. Effects of folic acid and vitamin b12 supplementation on cognitive impairment and inflammation in patients with alzheimer's disease: a randomized, single-blinded, placebo-controlled trial. J Prev Alzheimers Dis. (2021) 8:249–56. 10.14283/jpad.2021.2234101780

[14] Chen H Liu S Ji L Wu T Ji Y Zhou Y. Folic acid supplementation mitigates alzheimer's disease by reducing inflammation: a randomized controlled trial. Mediators Inflamm. (2016) 5912146. 10.1155/2016/5912146 27340344 PMC4909909

[15] Menon S Lea RA Roy B Hanna M Wee S Haupt LM. Genotypes of the MTHFR C677T and MTRR A66G genes act independently to reduce migraine disability in response to vitamin supplementation. Pharmacogenet Genom. (2012) 22:741–9. 10.1097/FPC.0b013e3283576b6b 22926161

[16] Di Rosa G Attinà S Spanò M Ingegneri G Sgrò DL Pustorino G. Efficacy of folic acid in children with migraine, hyperhomocysteinemia and MTHFR polymorphisms. Headache. (2007) 47:1342–4. 10.1111/j.1526-4610.2007.00932.x 17927652

[17] Yang Y Ligthart L Terwindt GM Boomsma DI Rodriguez-Acevedo AJ Nyholt DR. Genetic epidemiology of migraine and depression. Cephalalgia Int J Headache. (2016) 36:1097–25. 10.1177/0333102416638520 26966318

[18] Zhuan Z Ying O. Inference of MTHFR polymorphisms in migraine. J Clin Neurol. (2015) 28:3.

[19] Shiyin Z. Change of plasma homocysteine in migraine patients. Clin J Med Officers. (2002) 30:3. 10.3969/j.issn.1671-3826.2002.01.007

[20] Feng G Zhanxiu R Qiu H Shanshan S Meixi J. The contents and correlation studies of HCY and CRP in plasma of migraine in attack stage. J Apoplexy Nervous Dis. (2015) 32:3.

[21] Shuowen W. Prevention of folate deficiency with 5-methyl-tetrahydrofolate. Int J Pediatr. (2020) 47:4. 10.3760/cma.j.issn.1673-4408.2020.10.01132868164

[22] van der Put NMJ Gabreels F Stevens EMB Smeitink JAM Trijbels FJM Eskes T. A second common mutation in the methylenetetrahydrofolate reductase gene: An additional risk factor for neural-tube defects? Am J Hum Genet. (1998) 62:1044–51. 10.1086/301825 9545395 PMC1377082

[23] Min W Xiaobin C Jing L Gang L. *MTHFR* gene C677T polymorphism and migraine suscepitibility among Asian populations: a meta analysis Chongqing Med. (2020) 49:6. 10.3969/j.issn.1671-8348.2020.12.02435125689

[24] Lippi G Mattiuzzi C Meschi T Cervellin G Borghi L. Homocysteine and migraine. A narrative review. Clin Chim Acta. (2014) 433:5–11. 10.1016/j.cca.2014.02.028 24613517

[25] Yan P. The clinical significance of change of serum Hcy in patients with migraine. Chin J Tissue Eng Res. (2004) 8:92–3. 10.3321/j.issn:1673-8225.2004.01.047

[26] Lea R Colson N Quinlan S Macmillan J Griffiths L. The effects of vitamin supplementation and MTHFR (C677T) genotype on homocysteine-lowering and migraine disability. Pharmacogenet Genom. (2009) 19:422. 10.1097/FPC.0b013e32832af5a3 19384265

[27] Sherry ST Kholodov M Baker J Phan L Smigielski EM Sirotkin K. dbSNP: the NCBI database of genetic variation. Nucleic Acids Res. (2001) 29:308–11. 10.1093/nar/29.1.308 11125122 PMC29783

[28] Wang X Fu J Li Q Zeng D. Geographical and ethnic distributions of the MTHFR C677T, A1298C and MTRR A66G gene polymorphisms in chinese populations: a meta-analysis. PLoS ONE. (2016) 11:e0152414. 10.1371/journal.pone.0152414 27089387 PMC4835080

