# Supplementary material for: MTHFR polymorphism's influence on the clinical features and therapeutic effects in patients with migraine: An observational study
Source: Front Neurol. 2022 Dec 23;13:1074857. doi: 10.3389/fneur.2022.1074857 (PMC9816401; doi:10.3389/fneur.2022.1074857)
Supplement: Supplementary file 1 [file Table_1.docx]

**Supplemental Table 1. Clinical characteristics of migraine patients at baseline**

| Variables |  | N (%) |
| --- | --- | --- |
| Accompanying symptoms | Dizziness | 18 (13.3) |
|  | Nausea | 50 (37.0) |
|  | Vomiting | 50 (37.0) |
|  | Photophobia | 23 (17.0) |
|  | Phonophobia | 3 (2.2) |
|  | Sweating | 1 (0.7) |
|  | Blurred vision | 1 (0.7) |
| Pain aggravating factors | Staying up late | 4 (3.0) |
|  | Cold | 5 (3.7) |
|  | Nervousness and psychological stress | 9 (66.7) |
|  | Inadequate sleep and rest | 14 (10.4) |
|  | Neck and shoulder activities | 8 (5.9) |
|  | Tiredness | 16 (11.8) |
|  | Posture change | 3 (2.2) |
|  | Menstruation | 19 (14.1) |
|  | Bright light | 1 (0.7) |
|  | Weather | 1 (0.7) |
| Pain relief factors | Sleep and rest | 20 (14.8) |
|  | Supine position | 10 (7.4) |
|  | Massage | 1 (0.7) |
| Surgical procedures | Extracorporeal shock wave | 30 (22.2) |
|  | Stellate ganglion block | 18 (13.3) |
|  | Occipital nerve block | 18 (13.3) |
|  | Pulsed radiofrequency of occipital nerves | 13 (9.6) |
|  | Pulsed radiofrequency of C2 dorsal root ganglia | 8 (5.9) |
|  | Neurolysis of peripheral nerve | 7 (5.2) |
|  | Muscle release operation of trapezius | 5 (3.7) |
|  | Supraorbital nerve block | 4 (3.0) |
|  | Pulsed radiofrequency of bilateral greater occipital nerve | 4 (3.0) |
|  | Muscle release operation of temporalis | 3 (2.2) |
|  | Cervical facet joint block | 2 (1.5) |
